# Benefits and limits of biological nitrification inhibitors for plant nitrogen uptake and the environment

**DOI:** 10.1038/s41598-024-65247-2

**Published:** 2024-07-01

**Authors:** Christian W. Kuppe, Johannes A. Postma

**Affiliations:** 1 Institute of Bio- and Geosciences-Plant Sciences (IBG-2), Forschungszentrum Jülich GmbH, 52425 Jülich, Germany; 2 Faculty 1, RWTH Aachen University, Aachen, Germany

**Keywords:** Rhizosphere model, Bacteria, NUE, N leaching, BNI exudation, Plant sciences, Plant breeding, Natural variation in plants, Population dynamics

## Abstract

Plant growth and high yields are secured by intensive use of nitrogen (N) fertilizer, which, however, pollutes the environment, especially when N is in the form of nitrate. Ammonium is oxidized to nitrate by nitrifiers, but roots can release biological nitrification inhibitors (BNIs). Under what conditions does root-exudation of BNIs facilitate nitrogen N uptake and reduce pollution by N loss to the environment? We modeled the spatial–temporal dynamics of nitrifiers, ammonium, nitrate, and BNIs around a root and simulated root N uptake and net rhizosphere N loss over the plant’s life cycle. We determined the sensitivity of N uptake and loss to variations in the parameter values, testing a broad range of soil–plant-microbial conditions, including concentrations, diffusion, sorption, nitrification, population growth, and uptake kinetics. An increase in BNI exudation reduces net N loss and, under most conditions, increases plant N uptake. BNIs decrease uptake in the case of (1) low ammonium concentrations, (2) high ammonium adsorption to the soil, (3) rapid nitrate- or slow ammonium uptake by the plant, and (4) a slowly growing or (5) fast-declining nitrifier population. Bactericidal inhibitors facilitate uptake more than bacteriostatic ones. Some nitrification, however, is necessary to maximize uptake by both ammonium and nitrate transporter systems. An increase in BNI exudation should be co-selected with improved ammonium uptake. BNIs can reduce N uptake, which may explain why not all species exude BNIs but have a generally positive effect on the environment by increasing rhizosphere N retention.

## Introduction

The macronutrient nitrogen (N) is essential for plant metabolism amounting to 1–5% N of the total plant dry weight^1^. Greater N uptake promotes photosynthetic carbon fixation and plant growth. Plants take up nitrogen primarily as ammonium (${\text{NH}}_{4}^{ +}$) or nitrate (${\text{NO}}_{3}^{ -}$) from the soil solution. At the expense of energy, plants must reduce nitrate before nitrogen can be used to synthesize amino acids. While ammonium is energetically cheaper, plants can use ${\text{NH}}_{4}^{ +}$ and ${\text{NO}}_{3}^{ - }$, and usually have transporters that prefer the one over the other^2^. Nitrifying microorganisms, living in the influence sphere of the plant roots (rhizosphere), do the opposite and gain energy by converting ${\text{NH}}_{4}^{ +}$ to ${\text{NO}}_{3}^{ -}$ (nitrification). Studies often use the nitrifying bacteria *N. europaea* to test biological nitrification inhibitors (BNIs^)3,4^. Nitrate is at risk of becoming unavailable to the plant and polluting the environment by leaching or nitrogen oxide emissions during denitrification. According to a meta-analysis on cereal crop management, farmers fertilize with as much as three times the amount of N recovered in the crop yield, thus for every N two N are lost to the environment^5^. Ammonium binds promptly to the soil exchange complex. Thus, it has been proposed that N is better retained in the soil in the form of ammonium, and that prolonged retention might be achieved by inhibiting nitrification^6–8^. Synthetic nitrification inhibitors are commercially available and can be added to fertilizers, but they can potentially harm the environment and humans and have variable effectiveness^9^. They inhibit mostly ammonia-oxidizing bacteria and have less influence on ammonia-oxidizing archaea^10,11^. Soils vary in their composition of nitrifiers. Several plant species can exude BNIs from their roots^12–16^. For example, root exudates of *Sorghum bicolor* and *Brachiaria humidicola* contain BNIs^17,18^. Recently, BNIs have been found in exudates of major cereals, rice, maize, and wheat^16,19–21^. Exudation of BNIs has been suggested as a local and organic alternative for inhibiting nitrification, improving N uptake, and reducing environmental N pollution^22,23^. These BNIs are chemically diverse, including phenylpropanoids, quinones, benzoxazinoids, terpenes, and fatty alcohols^12,20,24^. In rice and wheat, genetic variation for BNI and nitrification promotion has been reported^16,19^. These results question the selection pressure for BNI exudation and the universal benefit of BNIs to plant N uptake. A more quantitative and mechanistic understanding of BNI functioning is necessary to understand their ecological and agronomic importance.

**Figure 1:**
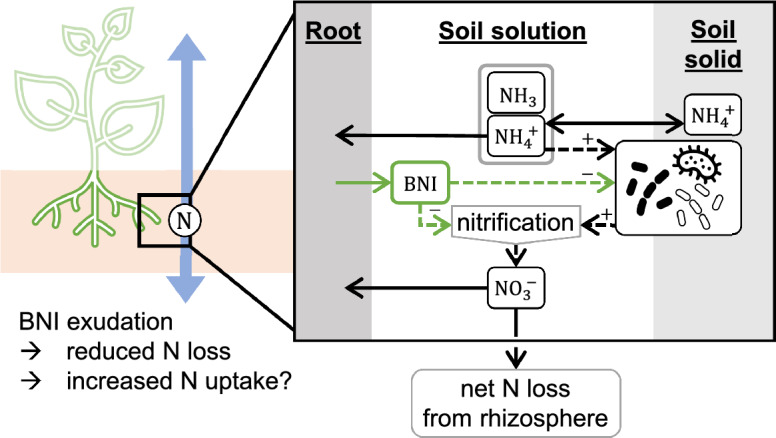
Schematic representation of the N-BNI rhizosphere model without indication of time and space: the influence of components is visualized by dashed lines. The root exudes BNIs (solid green arrow). BNIs influence both the specific growth of nitrifiers and nitrification rates (dashed green arrows). Double arrows denote rapid equilibria. Single solid arrows indicate fluxes and dashed single arrows reactions. ${\text{NH}}_{3}$ and ${\text{NH}}_{4}^{ +}$ are in a pH-dependent equilibrium (grey box).

We ask whether root-released nitrification inhibitors always promote soil N retention in the form of ammonium and N uptake (as sum ammonium and nitrate uptake over time) or whether these effects are evident only under specific rhizosphere conditions. The conditions we tested pertained to plant roots, soil physics, and nitrifiers. In particular, the uptake kinetics of the root can differ, and different soils can vary in the initial ammonium and nitrate concentrations, soil ammonium buffer power, and diffusion rates of BNIs. The nitrifier composition may differ in its growth rate and nitrification rate. Therefore, this study aims to give a theoretical basis for understanding the influence of different processes on the utility of BNIs. The present study answers our question through sensitivity analysis (results in Fig. 5) of a mechanistic spatiotemporal model of the rhizosphere (Fig. 1).

## Methods

We simulate spatio-temporal changes in concentrations of BNIs, ammonium, nitrate, and the population dynamics of nitrifiers in the rhizosphere. Symbols and references for parameter values are in Table 1.

**Table 1:** Parameters of the reference simulation, their description, and reference.

Symbol	Value	Unit	Description	References and comments
$\theta$	0.4	mL cm^−3^	Volumetric water content	Nitrification and minor denitrification^57^
$f$	0.25	-	Diffusion impedance factor	References^30,58^
${ v}_{0}$	$1\times {10}^{-7}$	cm s^−1^	Water flux into root	Reference^35^
${D}_{A}$	$1.58\times {10}^{-5}$	cm^2^ s^−1^	Self-diffusion constant of ${\text{NH}}_{4}^{ +}$	Linearly interpolated to 20°C and averaged over the two 0.1 M solutions^59^
${D}_{N}$	$1.64\times {10}^{-5}$	cm^2^ s^−1^	Self-diffusion constant of ${\text{NO}}_{3}^{ -}$	Linearly interpolated to 20°C and averaged over the two solutions and two concentrations, 0.1 M and 0.01 M, Ref.^59^
${D}_{\text{BNI}}$	$1\times {10}^{-7}$	cm^2^ s^−1^	Diffusion constant of exudate	Mid value of range $1\times {10}^{-9}$ to $1\times {10}^{-5}$
${ b}_{A}$	50	-	soil buffer power of ${\text{NH}}_{4}^{ +}$	Over two orders slower effective diffusion than nitrate, Ref.^60^
${ b}_{N}$	$\theta$	-	Soil buffer power of ${\text{NO}}_{3}^{ -}$	Non-sorbing
${ b}_{\text{BNI}}$	$\theta$	-	Soil buffer power of BNI	Non-sorbing
${ b}_{X}$	10	-	Soil buffer power of nitrifiers	In the lower range of the soil buffer power of the ${\text{NH}}_{4}^{ +}$ substrate
${A}_{{\ell},init}$	0.1	mM	Initial concentration of ${\text{NH}}_{4}^{ +}$ in soil solution	Reference^61^
${N}_{{\ell},init}$	0	mM	Initial concentration of ${\text{NO}}_{3}^{ -}$ in soil solution	
${X}_{{\ell},init}$	$3\times {10}^{4}$	cells cm^−3^	Initial cell density of nitrifiers in soil solution	See text
$l$	$9\times {10}^{-3}$	d^−1^	first-order rate constant for loss of nitrate to the environment	Calibrated to field-based N loss rate of 50% in the non-inhibited case (conservative estimate, see Refs.^5,53^)
Nitrification, growth and death of nitrifiers				
${ q}_{\text{max}}$	$1.5\times {10}^{-12}$	µmol s^−1^ cell^−1^	Oxidation rate	0.14–7 $\times {10}^{-3}$ fmol s^−1^ cell^−1^, Ref.^62^
$K$	0.1	mM (${\text{NH}}_{4}^{ +}$)	Saturation constant for oxidation	0.006 to 14 mM, Refs.^32,63^
${ \gamma }_{\text{max}}$	$2\times {10}^{-6}$	s^−1^	Specific growth rate of nitrifiers	0.52 $\times {10}^{-6}$ to 2.44 $\times {10}^{-5}$ s^−1^, Refs.^32,62,63^
${K}_{s}$	0.05	mM (${\text{NH}}_{4}^{ +}$)	Saturation constant for growth	0.051 to 0.07 mM, Ref.^32^
$Y$	$9\times {10}^{6}$	cells µmol^−1^	Yield constant of nitrifiers	0.7 $\times {10}^{6}$ to 17 $\times {10}^{6}$ cells µmol^−1^, Refs.^32,62^
$\delta$	$5\times {10}^{-2}$	d^−1^	First-order rate constant of microbial mortality	13.8 days half-life, Refs.^64,65^
Root: N uptake, exudation, and geometry				
${r}_{0}$	0.015	cm	Root radius	Reference^30^
${r}_{1}$	0.415	cm	Outer radius (from root center)	See text
${ F}_{\text{ex}}$	$7.9\times {10}^{-8}$	µmol cm^−2^ s^−1^	BNI exudation rate	See text
${ V}_{\text{max},A}$	$2.56\times {10}^{-6}$	µmol cm^−2^ s^−1^	Maximum ${\text{NH}}_{4}^{ +}$ uptake rate	Converted^19^
${ K}_{m,A}$	0.2869	µmol cm^−3^	Concentration of ${\text{NH}}_{4}^{ +}$ in soil solution (minus ${A}_{\text{min}}$) at which uptake rate is $1/2 {V}_{\text{max},A}$	Reference^19^
${ A}_{\text{min}}$	$1.5\times {10}^{-3}$	µmol cm^−3^	Uptake threshold of ${\text{NH}}_{4}^{ +}$	See ${N}_{\text{min}}$
${ V}_{\text{max},N}$	$4.69\times {10}^{-7}$	µmol cm^−2^ s^−1^	Maximum ${\text{NO}}_{3}^{ -}$ uptake rate	Converted^19^
${ K}_{m,N}$	0.1992	µmol cm^−3^	Concentration of ${\text{NO}}_{3}^{ -}$ in soil solution (minus ${N}_{\text{min}}$) at which uptake rate is $1/2 {V}_{\text{max},N}$	Reference^19^
${ N}_{\text{min}}$	$1.5\times {10}^{-3}$	µmol cm^−3^	Uptake threshold of ${\text{NO}}_{3}^{ -}$	0.3–9 $\times {10}^{-3}$ mM, Ref.^35^

### Model summary

We pose that the rooted soil domain can be represented by a root of unit length with an average associated soil volume (rhizosphere) given by the root length density (root length by soil volume). This approach to modeling the rhizosphere is elaborately described by Kuppe et al.^25^. The oxidation of ammonium to nitrite is assumed to be the rate-limiting process of nitrification, and nitrite accumulation is not considered. Therefore, and in agreement with other models^26–28^, nitrification is modeled as ammonium conversion to nitrate as a net-rate-function concerning a lumped group of nitrifiers in the rhizosphere model. This does not take the diversity of nitrifiers into account, but already goes beyond existing models by modeling the population dynamics and fits most experiments, which often only include *N. europaea*^3^. Principally, other sources of N could be ammonification or heterotrophic nitrification (from organic N to ${\text{NO}}_{3}^{ -}$). Heterotrophic nitrification occurs, however, especially in acidic soils where nitrification is anyway slow^4,29^. Also, dying microorganism can form a new organic N pool for ammonification. For the net effect of inhibition, we assumed the N retained in microbial biomass not becoming plant-available during the season, and we considered autotrophic nitrification only. Thereby, various forms of ammonification are assumed slower than the effect of BNIs. Consistently, the model has an initial ammonium input but no other sources of inorganic ammonium or nitrate production. All those processes are captured in the net loss and uptake terms.

Root-released BNIs can reduce both the oxidation rate of ammonium to nitrate and the growth rates of the nitrifier population. Roots can take up both ammonium and nitrate through their—by root hairs expanded—surface area, whereas the nitrifiers incorporate only ammonium into their biomass. Nitrifiers use ammonium for both the construction of proteins (immobilization) and for the conversion to nitrate (nitrification). Ammonium is the representative state variable in the model since ammonia and ammonium are assumed to be in equilibrium. Michaelis–Menten kinetics describe the uptake of ammonium and nitrate by the root simplified for all types of transporters^19^. Uptake of ammonium and oxidation to nitrate by nitrifiers are also saturating functions. The nitrate loss rate from the rhizosphere depends linearly on the nitrate concentration and represents the net loss of the whole soil domain without considering heterogeneity at a larger scale. Rhizosphere dynamics of 150-days were simulated, representing a typical growing season.

Plant-N uptake can be expressed relative to the amount of N in the soil and then is typically named nitrogen use efficiency (NUE) or N recovery. We define an average rhizosphere NUE for the rooted zone in a field as

1 $${\text{NUE}} = \frac{{{{\text{uptake NH} }_{4}^{ + } + {\text{uptake NO}}_{3}^{ - } \, (\upmu {\text{mol}})}}}{{{{\text{total initial inorganic rhizosphere N}} \, (\upmu {\text{mol}})}}},$$

We define a relative N loss to the environment (RNL) per initial amount of N in the rhizosphere as

2 $${\text{RNL}} = \frac{{{{\text{lost NO}}_{3}^{ - } \,\,{\text{from rhizosphere}} \, (\upmu {\text{mol}})}}}{{{{\text{total initial inorganic rhizosphere N}} \, (\upmu {\text{mol}})}}},$$

which is an average net N loss from the rooted zone in a field. We used NUE and RNL to evaluate the effect of BNIs on plant N uptake and N loss to the environment.

### Model development

#### Ammonium and nitrate transport-reaction equations

The changes in ammonium, *A*, and nitrate, *N*, concentrations in rhizosphere soil are described 1D radially (*r*) by ion transport in soil and reactions with the soil, root, and nitrifiers as

3 $$\frac{\partial A}{\partial t}=\frac{1}{r}\frac{\partial }{\partial r}\left(r{D}_{A}f\theta \frac{\partial {A}_{{\ell}}}{\partial r}+{v}_{0}{r}_{0}{A}_{{\ell}}\right)-{I}_{A} \, p(t)-\frac{1}{Y}\gamma X-qX+a,$$

4 $$\frac{\partial N}{\partial t}=\frac{1}{r}\frac{\partial }{\partial r}\left(r{D}_{N}f\theta \frac{\partial {N}_{{\ell}}}{\partial r}+{v}_{0}{r}_{0}{N}_{{\ell}}\right)-{I}_{N} \, p(t)+qX+l{N}_{{\ell}},$$

where $\partial A/\partial t={b}_{A} \partial {A}_{{\ell}}/\partial t$ is expressed in solution concentration ${A}_{{\ell}}$ with the soil buffer power defined as a constant, parameterized by initial concentrations, ${b}_{A}=dA/{dA}_{{\ell}}$; same for nitrate with ${b}_{N}=\theta$ (Table 1). The diffusion in pure water is ${D}_{A}$, ${D}_{N}$, multiplied by the soil impedance factor $f$ and the volumetric water content $\theta$. Radial water flux velocity to the root is modeled as a dynamic equilibrium by ${v}_{0}{r}_{0}$. The uptake of ammonium and nitrate by the root is modeled via sink terms in Eqs. (3) and (4) for the root hairs, ${I}_{A}$, ${I}_{N}$, and flux-boundary conditions at the root surface. The boundary and initial conditions are shown for nitrate (equivalent for ammonium). The flux at the root surface is described by (inner-boundary condition at $r={r}_{0}$)

5 $${D}_{N}f\theta \frac{\partial {N}_{{\ell}}}{\partial r}+{v}_{0}{N}_{{\ell}}=\frac{{V}_{\text{max},N}\left({N}_{{\ell}}-{N}_{\text{min}}\right)}{{K}_{m,N}+{N}_{{\ell}}-{N}_{\text{min}}}\hspace{0.17em}p(t).$$

The flux at the mid-distance to a (mirrored) neighboring root segment is (zero-flux outer-boundary condition at $r={r}_{1}$)

6 $${D}_{N}f\theta \frac{\partial {N}_{{\ell}}}{\partial r}+\frac{{v}_{0}{r}_{0}}{{r}_{1}}{N}_{{\ell}}=0.$$

The initial concentrations in soil solution are constant, $${A}_{{\ell},{\text{init}}}$$ and $${N}_{{\ell},{\text{init}}}$$, at $$t=0$$. The nitrifier population density in soil, $$X$$, is described below. The arrival of the root ($${t}_{R}=14)$$ is described by switching $$p(t)=0$$ to 1 when $$t\ge {t}_{R}$$. Thus, intake of ammonium by root and root hairs is described by the sink-term $${I}_{A}(r)$$, if present at distance $$r$$. Root hair uptake causes a rapidly forming diffusion profile, calculated as in Ref.^30^.

The nitrifier yield constant is *Y,* which represents an average C:N ratio over the functional group of nitrifiers because it relates the units of $X$ to ${A}_{{\ell}}$. Nitrogen loss to the environment is ${lN}_{{\ell}}$. The net ammonification rate is $a=0$ for simplification.

Ammonium concentration in soil solution, ${A}_{{\ell}}$, is the substrate that is enzymatically oxidized by nitrifying bacteria, which use the energy for life processes^31^. Thus, the relative nitrification rate is

7 $$q\!\left({A}_{{\ell}}\right)={q}_{\text{max}}\frac{{A}_{{\ell}}}{K+{A}_{{\ell}}}\cdot {f}_{\text{in}}\!\left({\text{BNI}}_{{\ell}}\right),$$

where the saturation constant for oxidation is *K*, and maximum oxidation rate constant is ${q}_{\text{max}}$. For the sake of parsimony, the modeled rates did not vary explicitly with temperature or water (the rates are varied directly in the sensitivity analysis), and the N in microbial biomass did not become plant-available during the season. BNIs may reduce the oxidation by a factor between 0 and 1, which is modeled as function, ${f}_{\text{in}}$, described below, which depends on the time–space distribution of the BNI-concentration in soil solution, ${\text{BNI}}_{{\ell}}$.

#### Biological nitrification inhibition by root exudates

**Figure 2:**
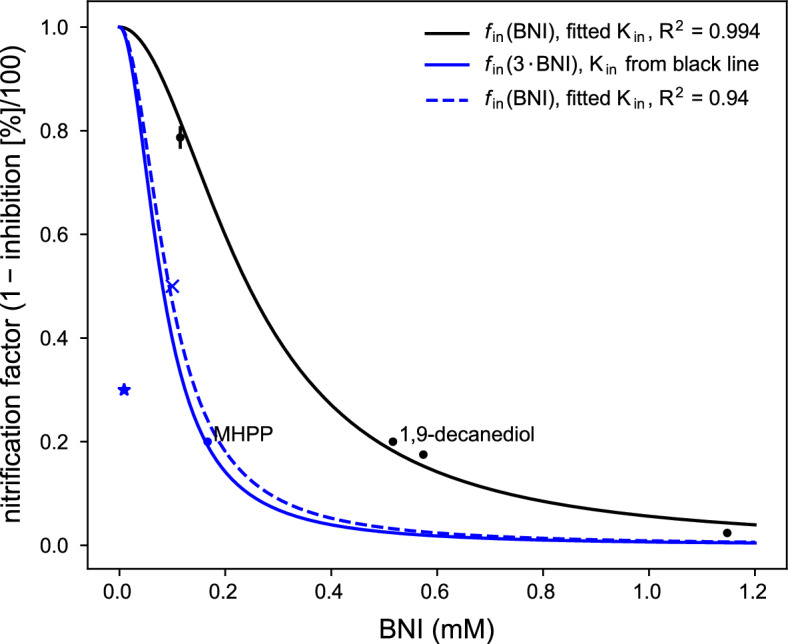
Nitrification inhibition, exemplified by the isolated BNI compounds 1,9-decanediol, black, and methyl 3-(4-hydroxyphenyl) propionate (MHPP), blue; from data by Sun et al.^19^, `dots', Subbarao et al.^6^, ‘x’, and Zakir et al.^17^, ‘star’. Fitting function: ${f}_{\text{in}}\!\left({\text{BNI}}\right)={\text{K}}_{\text{in}}/({\text{K}}_{\text{in}}+{\text{BNI}}^{2})$. The inhibition by isolated MHPP is about 2.5–3 times stronger than 1,9-decanediol in assay. The ${\text{K}}_{\text{in}}$ for the two solid lines was ≈ 0.0595 mM^2^. To obtain a first approximation for modeling inhibition, we used ${\text{K}}_{\text{in}}=0.00884$, dashed line.

In chemolithoautotrophs, the rate-limiting step of the nitrification pathway is mostly the first step by the enzyme ammonia monooxygenase^12,13^. Thus, if oxidation is described by a Monod equation, inhibition could be described in different ways: (1) using steady-state enzyme-inhibition kinetics for oxidases; or (2) empirically by scaling the limit ${q}_{\text{max}}$ or (3) by reducing the slope via $K$. Since studies on BNIs give values for the fractional reduction of nitrification the Monod equation (approach 2) was scaled with a fitted reverse saturation function (between one and zero). The function

8 $${f}_{\text{in}}\!\left({\text{BNI}}\right)=\frac{{\text{K}}_{\text{in}}}{{\text{K}}_{\text{in}}+{{\text{BNI}}}^{2}}$$

was fitted against inhibition data from literature (Fig. 2). The function *f*_in_ is unit-less and applied to $q$ and $\gamma$ to inhibit both growth and conversion to nitrate^12^.

The transport equation in the rhizosphere and accompanying initial-boundary conditions for the BNI concentration are

9 $${b}_{\text{BNI}}\frac{\partial {\text{BNI}}_{{\ell}}}{\partial t}=\frac{1}{r}\frac{\partial }{\partial r}\left(r{D}_{\text{BNI}}f\theta \frac{\partial {\text{BNI}}_{{\ell}}}{\partial r}+{v}_{0}{r}_{0}{\text{BNI}}_{{\ell}}\right)+{E}_{\text{h}}\hspace{0.17em}p(t)-{l}_{\text{deg}}\text{BNI}$$

10 $${D}_{\text{BNI}}f\theta \frac{\partial {\text{BNI}}_{{\ell}}}{\partial r}+{v}_{0}{\text{BNI}}_{{\ell}}={F}_{\text{ex}}\hspace{0.17em}p(t) \qquad \text{at }r={r}_{0},t>0$$

11 $${D}_{\text{BNI}}f\theta \frac{\partial {\text{BNI}}_{{\ell}}}{\partial r}+\frac{{v}_{0}{r}_{0}}{{r}_{1}}{\text{BNI}}_{{\ell}}=0 \qquad \text{at }r={r}_{1},t>0$$

12 $${\text{BNI}}_{{\ell}}=0 \qquad \text{at }t=0,$$

where $p(t)=1$ for $t\ge {t}_{R}$, else $p(t)=0$, and $\text{BNI}={b}_{\textrm{BNI}}{\text{BNI}}_{{\ell}}$. As with uptake, the exudation starts at the arrival time of the root, ${t}_{R}=14.$ ${F}_{\text{ex}}$ is the exudation rate at the root surface *r*_0_. The source-term of BNI from root hairs ${E}_{\text{h}}$ is described over the volumetric root hair surface area: ${E}_{\text{h}}(r)={\text{A}}_{\text{h}}(r){F}_{\text{ex}}$, for *r* not larger than the average root hair length and ${\text{A}}_{\text{h}}\!\left(r\right)$ describes the root hair surface per volume depending on the distance *r*. Over time, microbial degradation of BNI is possible but set to zero (i.e., not simulated): ${l}_{\text{deg}}=0$. Other factors can inhibit oxidation, like too much or little oxygen^32^. This study excludes these effects and assumes that limited oxygen and carbon dioxide do not further inhibit nitrification. For example, similar models investigated the inhibition of denitrification by oxygen in wetlands in the rhizosphere^26^, and on the ecosystem scale of the savanna, using ODE systems^27,28^.

#### Nitrifier population

The previous partial differential equations are discretized such that the rhizosphere domain is divided radially into compartments. The density of microorganisms is modeled as ordinary differential equation (ODE) system. An ODE for each spatial compartment ($i=1,...,n$) from the numerical discretization of the substrate, resulting in a spatial gradient of nitrifier cell density

13 $$\frac{\partial {X}_{i}}{\partial t}=\left(\,\gamma\!\left({A}_{{\ell},i}\right)-\delta\,\right){X}_{i},$$

where ${{X}}$ is a vector with entries ${X}_{i}=X({r}_{i},t)$ and represents the total cell density of nitrifiers in soil defined as $X={b}_{X}{X}_{{\ell}}$, with soil buffer power of nitrifiers, ${b}_{X}$, and nitrifier density in soil solution, ${X}_{{\ell}}$. Growth ($\gamma$) and death ($\delta$) hold for ${{X}}$, that means nitrifiers attached on soil particles are active, but they depend on the substrate in the solution ${A}_{{\ell}}$. The relative growth rate of the microbial population is described by

14 $$\gamma\!\left({A}_{{\ell}}\right)={\gamma }_{\text{max}}\frac{{A}_{{\ell}}}{{K}_{s}+{A}_{{\ell}}}\cdot {f}_{\text{in}}\!\left({\text{BNI}}_{{\ell}}\right),$$

where BNIs reduce the growth by factor ${f}_{\text{in}},$ ${\gamma }_{\text{max}}$ is the maximum growth rate and ${K}_{s}$ is a saturation constant for growth of nitrifiers. The microbial death rate coefficient is denoted by $\delta$, and is usually first-order, therefore, set constant. Motility of nitrifiers was not important to NUE and RNL^33^, so that Eq. (13) is modeled as ODE system, where population sizes are still spatially distinct.

### Model parameterization

The parameter values are within ranges based on the literature (Table 1). Most BNI research seems to focus on *Poaceae*, especially crops like rice, maize, and sorghum. Our reference parameterization is thus strongly based on data from these species, particularly rice. We regard our parameterization, however, species indifferent, especially because our sensitivity analysis covers a large parameter space, in which the niches of many species and genotype might be represented.

#### Nitrification, cell density and growth of nitrifiers

Parameters on specific oxidation can differ in their unit: relating to cells, *amoA* genes, or gram carbon as microbial biomass, and some publications did not normalize for bacterial concentrations but give rates of nitrate production in soil. We used the maximum oxidation rate constant based on data on cell densities of nitrifiers. We used default values approximately in the middle of reported minimum and maximum values for *q*_max_, *K*, ${\gamma }_{\text{max}}$, *K*_*s*_, and *Y*. The reference value of the initial cell density of nitrifiers was calibrated to agree with the oxidation rate coefficients and ammonium concentrations.

#### Exudation of BNIs

**Figure 3:**
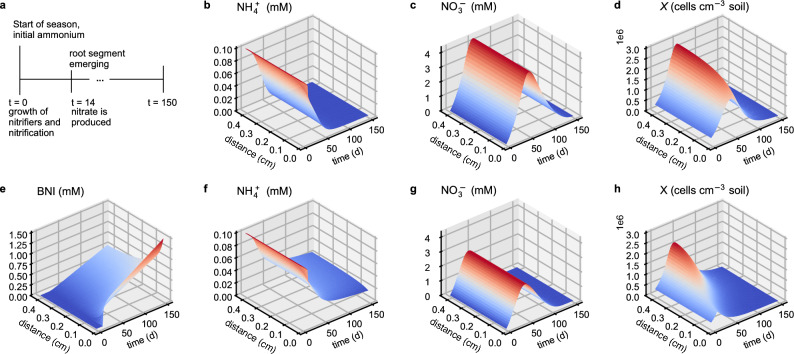
Simulation results of the reference simulation (parameter values in Table 1) without (top) and with (bottom) the exudation of BNIs. Graphs show the spatial–temporal dynamics in ammonium (**b**,**f**), nitrate (**c**,**g**), and BNI (**e**) concentrations in soil solution and nitrifier cell densities in soil (**d**,**f**). The distance is radial to a representative root segment of unit length (root center at distance r = 0) and the colors match the contour lines. (**a**) Timeline of simulated season (150 days): nitrification starts at *t* = 0, uptake, and exudation by the root segment at *t* = 14 days. Uniform growth of nitrifiers for *t *< 14 days.

Chemically BNIs are a mixture of exudates consisting of various compounds, some hydrophobic others hydrophilic. Synergistic behavior of those different BNIs is suggested but not yet confirmed^6,13^. Hence, BNIs can differ in their solubility in water and, therefore, the apparent diffusion rates. Hydrophobic BNIs may concentrate at the rhizoplane, sticking to the root hair tips or main surface but may also travel in soil, whereas hydrophilic BNIs may diffuse and maybe even leach as this was found problematic with synthetic inhibitors. For the reference simulation, we used a moderate diffusion rate coefficient of BNIs in liquid, *D*_BNI_, which is between the self-diffusion of water (order 1e−5 cm s^−1^) and very slow diffusion (1e−9 cm s^−1^). Note for the analysis that this *D*_BNI_ is similar to the effective diffusion of BNIs in the soil solution (Fig. 3e) as we excluded the explicit modeling of adsorption of BNIs to the soil solid by setting the BNI soil buffer power to $\theta$.

Often, rates for exudation and maximum uptake rate coefficients are given as g per g root dry weight (DW) per unit time. Since such rates are estimated over the whole root system, they need to be understood as root system averages and do not account for local responses. Nevertheless, we used an average gram per root surface area factor to convert data and account for root diameter. Average factors for gram DW per rice root volume are calculated from the data^30^. The average g (DW) cm^−2^ values are 0.00027, 0.00022, and 0.00034 for day 7, 14, and 21 after emergence, respectively. The values corresponding to g (DW) per root volume are 0.045, 0.038, and 0.065 for the same time points. For the used root radius and root hair parameters, this is 0.076 g per root volume, which is a plausible value^34^ as an average conversion factors that does not account for root structure (fine root vs. thick roots).

**Figure 4:**
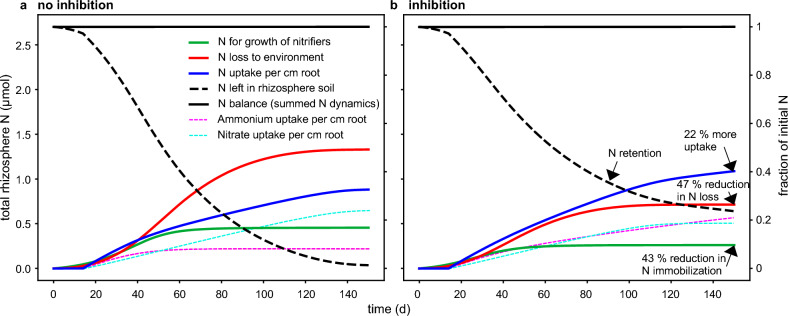
Time dynamics in the size of N contribution for simulations without (**a**) and with (**b**) inhibition. When BNIs are exuded, N uptake is increased, and N loss and immobilization are reduced. At the end, there is nitrogen left in the rhizosphere, suggesting fertilization can be reduced. The dashed lines show inorganic N retained in the rhizosphere due to adsorption. Parameter values are in Table 1. Distinction between uptake by hairs and main root surface is shown in Supplemental Fig. S2.

The exudation rate was set concerning 10.8 mg g^−1^ root day^−1^^13,17^ and converted to cm^−2^. However, when comparing the BNI concentration near the root and the inhibition function ${f}_{\text{in}}$, this rate might be an upper limit. Therefore, we reduced this rate for the water-soluble exudate compound by one-third (the values for MHPP served as approximation for the inhibition). The root and root hairs exude BNIs, gives a concentration range appropriate for ${f}_{\text{in}},$ (Fig. 4e). The reported values of the hydrophobic BNI were over four orders of magnitude lower^19^. The mobility of BNIs, thereby, remains uncertain.

#### Root and root hairs

Root and root hair geometries are based on data from L-type lateral roots of rice^30^: a root hair number on the root surface per unit length of 700 cm^−1^, a root hair radius of 0.0008 cm, and an average root hair length of 0.015 cm. However, the root radius can also represent a small lateral root of another species. With a slightly larger mid-distance to neighboring roots, *r*_1_, and no other lateral roots branching into the rhizosphere of unit length, the root system has a representative root length density (1.85 cm cm^−3^).

#### Root uptake kinetics

Uptake is modeled with Michaelis–Menten kinetics for the ammonium and nitrate concentration ranges, including minimum concentrations for uptake (${A}_{\text{min}},{N}_{\text{min}}>0$, Table 1). Data on the uptake kinetics of the nitrification inhibiting six-week-old rice seedling, Wuyunjing7 (WYJ7), without the minimum concentration for uptake is used^19^. A minimum concentration for uptake means in the model that the plant can lose nutrients if the concentration at the root surface drops below that minimum concentration. Since the maximum uptake rates were in units per gram root DW, we estimated the uptake kinetics with the same gram-to-root surface area conversion factor described above for the BNI exudation. Note that WYJ7 inhibits nitrification, and Wuyujing3 (WYJ3) does not; WYJ3 even promotes nitrification^19,21^.

### Simulation

**Figure 5:**
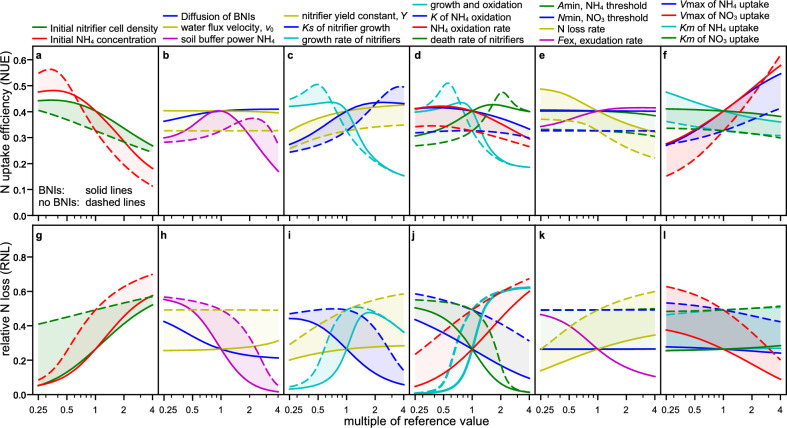
Sensitivity of N uptake efficiency (first row) and relative loss (second row) to variation in parameter values. The shading indicates the difference between simulations with inhibition by exudation (solid lines) and without (dashed lines). The x-axes indicate the change in the parameter value relative to the value of the reference simulation. Note that the x-axis is logarithmic, and a 1:1-line is not straight in a semi-log-plot. The relative change in sensitivity (steepness of curves) can be compared between all sub-plots since the y-axes of each sub-plot have the same relative scaling to their reference simulation without inhibition. Note that the initial N concentration varies only in (**a**) and (**g**) in [1.25; 20] mM ammonium in soil, which is buffered, i.e., [0.025; 0.4] mM ammonium in the plant available soil solution and thereby non-toxic to plants. For varying the soil buffer power, we adjusted the initial concentration in soil solution to keep the total ammonium constant in (**b**) and (**c**). BNIs increase uptake, except if ${\text{NO}}_{3}^{ -}$ concentration is low, or highly sorbing, ${\text{NO}}_{3}^{ -}$ uptake rate is high or ${\text{NH}}_{4}^{ +}$ uptake rate is low, nitrifiers grow slowly, or their death rate is high, or both nitrifier growth and oxidation are slow. However, if the death rate is too high, the reduction of nitrifier population is too fast such that nitrifier and their inhibition does not matter (**d**,**j**). BNIs always reduce RNL (**g**–**l**).

The simulations were performed with and without inhibition by exudation of BNIs and the reference parameter values in Table 1. The simulation duration was 150 days, representing a typical life cycle of an annual plant, and the first 14 days were considered without root as an initial period of nitrification when the root is not established yet. Hence, until day 14, the nitrifiers can grow freely, and if enabled, BNI exudation starts on day 14 (Fig. 3a). In the sensitivity analysis, the initial N concentration was held constant except for Fig. 5a,b. That means for the change in the soil buffer power, the initial concentration in soil solution was changed according to

15 $${A}_{{\ell},{\text{init}},{c}_{b}}={A}_{{\ell},{\text{init}}}/\left({c}_{b}\cdot {b}_{A}\right),$$

where ${c}_{b}$ is a scalar expressing the relative change in ${b}_{A}$. The total initial N changed for changing initial ammonium concentrations.

The sensitivity of NUE and RNL to changes in parameter values was analyzed by varying the parameter values relative to their default over a 16-fold change, four times smaller or larger than the reference values.

Compared to literature values^35–38^, our nitrification rates of the reference simulation are within the range but at the lower end, calculated from the slope of the first 30 simulated days. For example, Mohanty et al.^38^ applied three doses of 10 mM NH_4_-N to a soil with 225 mg kg^−1^ available N, and reported a range of potential nitrification rates as produced 0.49–0.65 mM ${\text{NO}}_{3}^{ - }$ g^−1^ soil day^−1^. In agricultural soils, nitrification rate-ranges of 1–5 and 1–34 mg N kg^−1^ soil day^−1^ can be found^36^. Soil conditions, sorption, and initial concentration, influence nitrification rates dramatically.

The model was solved by a backward differentiation formula with variable order from 1 to 5 and a spatial step size of $\Delta r$ = 2e−3 cm^39,40^. We used relative and absolute tolerances of 1e−3 and 1e−6, respectively, for the solute concentrations and cell densities of nitrifiers as well as the cumulative N uptake by the root and nitrifiers (Fig. 4). The time stepping was adaptive and starting and maximum time steps were set to 1e−8 and 0.1 days, respectively.

## Results

First, a reference simulation parameterized with values in ranges found in the literature was established (Table 1, Fig. 3). With these reference values, the model simulated that BNIs increased total N uptake by the root segment by almost 22%, raising the rhizosphere N use efficiency (Eq. 1, NUE) from 33 to 40% (Fig. 4 blue lines and Fig. 5 for multiplier = 1). The relative N loss (Eq. 2, RNL) decreased by 47% (from 49 to 26%), and the inorganic N converted to microbial biomass was reduced by over 43%.

Next, we varied soil, microbial, and plant conditions to show when BNI exuding plants have an advantage. This sensitivity analysis shows the NUE and RNL outcome over a 16-fold change in parameter values (Fig. 5). In Fig. 5, the inhibition gap is the difference between the results without (dashed lines) and with inhibition (solid lines). The difference between N uptake with and without inhibition can swap signs, indicating inhibition affected uptake negatively. In all scenarios, BNIs reduced RNL, although this benefit of BNIs varied strongly. NUE was often improved by BNIs, but not always. We summarize the results by looking at the governing processes that influence ammonium and nitrate uptake by the root.

### BNIs are beneficial when ammonium concentrations are large

The initial ammonium concentration determines whether BNIs increase or decrease NUE (Fig. 5a, red lines, also supplemental Fig. S1). For example, the same N uptake as the reference simulation (without inhibition) can be reached with 25% less initial ammonium when the root exudes BNIs and nitrate loss over the season would be reduced by 70% (Fig. S1). Low initial ammonium concentration increases NUE in all conditions. The rhizosphere ammonium concentration is not depleted, with and without BNIs. However, inhibition of nitrate production by BNIs decreased total N uptake and NUE. A greater uptake was achieved when both nitrate and ammonium were available in the rhizosphere, such that both uptake transporter systems take up N at elevated rates (Pareto efficiency). Low initial ammonium decreases loss because nitrification is slow, and the plant takes up the produced nitrate before it is lost. In these scenarios, further inhibition of nitrification by BNI, on top of the intrinsic inhibition caused by the low initial ammonium concentrations, does not reduce loss further but reduces uptake of nitrogen. Inhibition affected N loss to a lesser extent (smaller gap) if the initial cell densities of nitrifiers were high (Fig. 5g).

### BNIs are beneficial when the adsorption of ammonium to the soil is low

When we changed the soil buffer power^25^ of ammonium (= dissolved/total ${\text{NH}}_{4}^{ +}$), we changed the initial concentration of ammonium in the soil solution proportionally to keep the total initial concentration constant. This soil buffer power can be increased by soil management, e.g., by addition of biochars^41^. A decrease in soil buffer power decreased N uptake by the plant and increased loss from the rhizosphere (without and with inhibition, Fig. 5b,h). Despite the larger initial concentration in the soil solution, ammonium was depleted faster with reduced soil buffer power. However, an increase in soil buffer power of ammonium decreased ammonium in solution and, consequently, nitrate production. Hence, compared with the reference simulation (see buffer powers just greater as the reference value, *b*_*A*_ = 50, for BNIs and more than double without BNIs), this nitrate concentration in the rhizosphere was depleted faster, and the root’s nitrate uptake system became under-utilized. Nitrifiers replenish the nitrate concentration in the rhizosphere when not inhibited. Hence, in soils that strongly adsorb ammonium, BNIs are not beneficial.

Sorption reduces the effective diffusion rates and thereby the concentration of BNIs throughout the rhizosphere. Slow effective diffusion of BNIs, which is the diffusion in soil accounting for sorption, volumetric water content, and tortuous pathways (here 0.25∙*D*_BNI_ = 2.5 × 10^−8^ cm^2^ s^−1^), causes BNIs to accumulate at the root surface, reducing their overall effectiveness across the rhizosphere (Fig. 5b,h, blue line).

The reference values for BNI exudation rate and soil buffer power resulted in near optimal NUE (Table 1, Fig. 4b,h, pink lines). Increasing the rate of BNI exudation, *F*_ex_, leads to saturation of the relative effectiveness of BNIs on uptake (here NUE of over 41%), which indicates a good balance in the reference simulation between metabolic cost of BNI exudation and its utility to increase N uptake. N uptake and loss were insensitive to variation in the water flux velocity, ${v}_{0}$, caused by the transpiration stream (Fig. 5b,h).

### BNIs are beneficial when the nitrifier population growth is neither fast nor slow

The effect of BNIs on NUE and RNL was sensitive to changes in the parameter values of nitrifier growth (*γ*_max_ and *K*_*s*_, Eq. (14); Fig. 5c,i, cyan and blue lines). Fast growth (high *γ*_max_ or low *K*_*s*_) led to the immobilization of ammonium, and the consequently larger nitrifier population oxidized the remaining ammonium rapidly. The resulting low ammonium concentrations limited nitrification, thereby, making BNIs redundant once exuded. When the ammonium concentration in the rhizosphere depleted, roots and nitrifiers competed for ammonium. This competition reduced the total N uptake by the plant. If the nitrifier population grew too slowly (e.g., *γ*_max_ ≤ 1.5 × 10^−6^ s^−1^), BNIs did not facilitate uptake either because nitrate production was insufficient to maintain total N uptake. Thus, both low and high nitrifier growth rates lead to BNIs being less effective.

The yield constant of nitrifiers, *Y* [cell µmol^−1^], relates the population size to ammonium concentrations and is associated with the C:N ratio in the nitrifier biomass. It inversely affects the ammonium uptake by nitrifiers in Eq. (3), and it cancels out the unit of the nitrifiers, here cells. The nitrifiers incorporate this N into their biomass while growing. The yield constant might vary for different nitrifier species. Changes in this parameter value influenced N loss more than N uptake by the root. The greater the yield constant, the greater NUE because there is slightly less ammonium taken up by nitrifiers and, therefore, more ammonium converted to nitrate, which was then available for uptake by the root. For small yield constants, the ammonium concentration depletes earlier. And for larger yield constants, more nitrate is produced, resulting in greater RNL.

### BNIs are beneficial when nitrifiers are persistent in soils

For death rates four times the reference value, there was no difference with or without exudation because nitrifiers died early (Fig. 5d,j, green lines) without contributing to the inorganic ammonium concentration. For variation in death and growth rates, uptake had optima, hence, the nitrifier population should not be too low, producing sufficient nitrate for uptake by the plant over the season, and not be too high, converting the ammonium too quickly to nitrate. E.g., the optima for death rates without BNIs was around 2.25 times the reference value and yielded an NUE of 47%. Those numbers are all accessible (see the data availability statement below).

### Uptake is insensitive to changes in oxidation rates

The nitrification rate, *q*, increases with increasing maximum oxidation coefficient, ${q}_{\text{max}}$, and decreasing saturation constant for oxidation, ${K}_{m}$, Eq. (7). Nitrogen uptake and loss were less sensitive to variation in nitrification rates than death rates (Fig. 5d,j, compare blue and red to green lines). Biological nitrification inhibitors function on both processes, but the greater sensitivity for death rates implies that control over the nitrifier population is the more important function. Increased death rates without BNIs exceeded the effect of BNIs because controlling the population by BNIs has a delay.

### BNIs decrease the sensitivity to nitrate loss rate

The sensitivity of NUE and RNL regarding changes in the first-order rate coefficient of N loss ($l$) is almost reciprocal to changes in the exudation rate. With BNI exudation, the RNL was more robust to nitrate loss. Biological nitrification inhibitors are slightly more effective for slower loss (Fig. 5e,k, yellow lines).

### Uptake kinetics of the root determine the efficacy of BNIs

Nitrogen uptake and loss were insensitive to changes in the minimum concentrations for uptake of ammonium and nitrate by the root, *A*_min_, *N*_min_, Eq. (5), but BNI efficacy was affected by the other kinetic parameters, ${V}_{\text{max}}$ and ${K}_{\text{m}}$. When the plant is able to take up nitrate very fast (large ${V}_{\text{max},N}$), inhibition did not facilitate uptake. The opposite is true for roots with fast ammonium uptake (large ${V}_{\text{max},A}$, Fig. 5f). The lines for the affinity constants ${K}_{{\text{m}},N}$ and ${K}_{{\text{m}},A}$ are less sensitive than ${V}_{\text{max},N}$ and ${V}_{\text{max},A}$. Thus, whether BNIs are beneficial for uptake depends on soil conditions as well as on the uptake transporters of the root. Taken together, these results indicate that the biggest gain in plant nitrogen uptake is expected by inhibiting growth of nitrifiers and, concurrently, by increasing plant ammonium uptake kinetics (for similar conclusion see^42^).

## Discussion

Since nitrogen is essential for plant growth and functioning, and intensive agriculture, with its highly fertilized soils, secures food supply but pollutes the environment with N, we asked if and under what conditions BNIs improve both NUE and RNL. BNI exudation benefits uptake often, but not in all scenarios. Our modeling results support previous suggestions that BNI exudation can increase NUE, especially when plants take up ammonium faster than nitrate, as is the case for many rice genotypes^19,43^.

The time dynamics of rhizosphere ammonium and nitrate concentrations ultimately determine the efficacy of BNIs for plant N uptake and N loss. Determining processes are (1) competition for nitrogen between plant roots and microbes, (2) the rate of depletion of ammonium, and (3) the utilization of the root’s nitrate transporters (synergistic uptake^13^). Low ammonium concentration lowers nitrification rates, and no further inhibition is necessary. The N uptake by the root is limited by uptake kinetics (*V*_max_, *K*_*m*_) and is maximal when both the ammonium and the nitrate transporters are saturated. This roughly happens when the N concentrations at the root surface are greater than 2–3 times *K*_*m*_. At larger ammonium concentrations, nitrification increases total N uptake by increasing the nitrate concentrations as long as the decrease in ammonium concentrations, caused by nitrification, does not decrease ammonium uptake by equal amounts. This relationship could explain why exudates of some rice varieties promote nitrification^19^.

BNI exudation has a metabolic cost to the plant and, as shown here, does not always increase total N uptake, which may explain why BNIs are not found in all species. In environments that are less favorable for the nitrifiers, the reduced growth and longevity make BNIs redundant. The same is true in low nitrate and ammonium environments, which result in an intrinsic inhibition by the low concentrations. Selection pressure in these environments might have been low. However, BNI production was found in species adapted to low-N conditions. This does not prove that BNIs are particularly beneficial in low N environments but might suggest that^13^. If so, it would contradict the simulation results and may need further investigation. Overall, we expect nitrification to be slow when ammonium concentrations are low, and thus, further inhibition of nitrification would be unnecessary. Increased initial ammonium concentrations typically occur under agricultural fertilized conditions, which makes the ability to release BNIs a breeding target^44^.

In some species, ammonium uptake causes an excess uptake of cations over anions, and this imbalance triggers BNI exudation^45–47^. However, instead of an ammonium dependent exudation, our model necessitates negative feedback to nitrate concentrations, because the root’s nitrate transport system can be under-utilized when BNIs exudation reduces nitrate production. If both ammonium and nitrate are sufficiently available to saturate uptake over the growth period, nitrification inhibition does not affect N uptake. Future experiments could test whether uptake-saturating ammonium concentrations promote, and low nitrate concentrations inhibit BNI exudation balancing uptake and investment by the plant. This feedback would also address the question if an exudate inhibits specifically^13^.

Exudates might reduce the enzymatic oxidation or the nitrifier population size. BNIs from tree roots slow down nitrifier growth, while those from wheat reduce ammonium oxidation^15,48^. Many experiments, however, do not distinguish death, growth, and oxidation. In bacteria assays, addition of the intermediate substrate hydroxylamine only partially rescued the nitrification process inhibited by BNIs^19,49^. This means either that not all enzymes were inhibited or that bacteria died, which is not distinguished by checking the resulting hydroxylamine. Thus, the inhibition of enzymes was proposed^6,18^, but that does not rule out other modes of action^12^, as fatty alcohols can potentially damage the cell membranes of bacteria^50^. Recent progress shows that specific BNIs can have specific mode of action, for example, Kaur-Bhambra et al.^51^ showed that a novel BNI, N-butyldodecane-1-amin, is specific to ammonia-oxidizers and does not affect other bacteria and Li et al.^11^ found that sorghum root exudates (as a whole) inhibited AOB but not AOA. Thus, screening criteria for BNIs may be further refined. Bacteria might be inactivated by the BNI and potentially killed (bactericidal). Our simulations indicate the latter would be more beneficial to the plant as it avoids N immobilization into the bacterial biomass. A bactericidal function, however, might affect other microbes as well, with unknown consequences for soil health and ultimately plant performance. As a first step, we suggest that assays of BNIs record besides the rate of nitrification also the population dynamics of the nitrifiers.

Our model assumes that initial ammonium concentration is converted by nitrifiers, such that nitrate is present at root emergence. This scenario may represent a broad range of starting conditions: application of urea or ammonium fertilizer and zero nitrate pre-season, or soil with a high mineralization rate after a cold or dry season. During the season, root length density increases such that the field can be defined as rhizosphere quickly. The sensitivity analysis, for example, for the nitrifier death rate parameter, affected the simulation over the whole period, whereas BNIs, which also affect the population dynamics, only became effective later during the simulation. Thus, NUE and RNL were more sensitive to changes in the parameter values than to BNIs and might indicate that synthetic nitrification inhibitors can be more effective as they work earlier. On the other hand, BNIs continue to be exuded throughout the season, whereas the synthetic ones must be degradable. This result indicates that both inhibitor forms (synthetic and biological) could be complementary. The rate of (urea) fertilizer availability due to different coatings can also decrease the nitrification rate over time^4^. BNIs and urease inhibitors could be co-applied to further decrease N loss in calcareous soils^41,52^. Since, in N-enriched environments NH_3_ emission was shown to be increased when nitrification was inhibited (synthetically) but the net effect was beneficial due to reduced inorganic N leaching, N_2_O emission, and NO emission^53^. Further, BNIs are supposed to act locally, and root zone applied fertilizer reduced NH_3_ volatilization^54^.

We kept the size of the rhizosphere constant, which implies a constant root length density. This assumption might be reasonable under ecological conditions and pastures with continuous ground cover over time. Annual crops are typically planted in fields, where root length density might vary over time strongly. Root length density typically increases with nitrate and ammonium availability, as plants grow faster^55^. Future studies will need to determine the importance of these scenarios for the efficiency of BNIs. However, because BNIs are chemically diverse with different molecule sizes and include hydrophilic (e.g., MHPP^3^) and hydrophobic BNIs (e.g., 1,9-decanediol^56^), we regard addressing current uncertainty in the diffusion coefficient and mode of action of BNIs as a higher priority. This study considers one type of BNI: whether certain combinations of slow or moderate, and fast effective diffusion rates of BNIs, which may be stable and non-stable BNIs, (spatially and temporally) complement each other needs to be proven. We took determined parameter values from published measurements, and the simulated rates of ammonium oxidation seem within the range of reported values for nitrate production and its inhibition. Still, much uncertainty remains to their precise values in relation to different environmental factors such as soil temperature and water content^35–37,50^.

At the end of the simulated season, there may be enough cells left to regrow and continue ammonium oxidation. Note that the nitrifier pool was not empty at *t* = 150 days. However, nitrifier recovery was not simulated and not the aim of this study.

In conclusion: BNI exudation should go hand in hand with enhanced ammonium uptake. Breeders may select for kinetic traits together with BNIs. Nitrifying microorganisms are competitors for ammonium, but they are necessary to produce some nitrate to maximize the use of both ammonium and nitrate uptake systems. Nitrogen uptake by the root is less sensitive to changes in oxidation rates of ammonium by nitrifiers. Controlling the population size of nitrifiers affects total N uptake more than controlling their nitrification activity. This result indicates that, for nitrifier-plant interaction and the effect on NUE, exudation should be bactericidal and not bacteriostatic—kill nitrifiers but not too many. However, some nitrification is needed to maintain nitrate concentrations.

An increase in BNI concentration reduces N loss but does not always increase N uptake. BNIs even decrease uptake when (1) the (initial) soil ammonium concentration is low, (2) the ammonium soil adsorption is high, (3) nitrate uptake is fast relative to ammonium uptake, or (4) the nitrifier population grows slowly, self-competes, or (5) declines fast. These cases imply that the utility of BNIs depends on soil type, and on the specific growth rates and activity of nitrifiers. Above a threshold, fertilization and BNIs gives more uptake or less fertilizer for same uptake.

Inhibition can only facilitate plant-N uptake and uptake efficiency when the inferred lower nitrate production has no effect on the nitrate uptake rate or is compensated by increased ammonium uptake. With inhibition, there is potentially longer sustained uptake of ammonium over time, but that cannot compensate for the reductions in uptake when the nitrate pool is depleted early. Nitrogen is a pollutant, and under the right conditions BNIs may avoid N loss while increasing NUE. Thereby, BNIs indirectly promote carbon fixation and crop production.

### Supplementary Information

Supplementary Figures.

## Data Availability

All model results are available in the data repository at figshare: Dataset 10.6084/m9.figshare.22737158.
