# Supplementary material for: Benefits and limits of biological nitrification inhibitors for plant nitrogen uptake and the environment
Source: Sci Rep. 2024 Jul 1;14:15027. doi: 10.1038/s41598-024-65247-2 (PMC11217430; doi:10.1038/s41598-024-65247-2)
Supplement: Supplementary file 1 — Supplementary Figures. [file 41598_2024_65247_MOESM1_ESM.pdf]

Supplemental Figures to "Benefits and limits of biological nitrification inhibitors for plant nitrogen uptake and the environment"

Christian W. Kuppe and Johannes A. Postma

Scientific Reports, 2024

List of Figures

S1 Influence of initial N in soil 2
S2 Cumulative uptake of nitrate and ammonium by root and root hairs 3

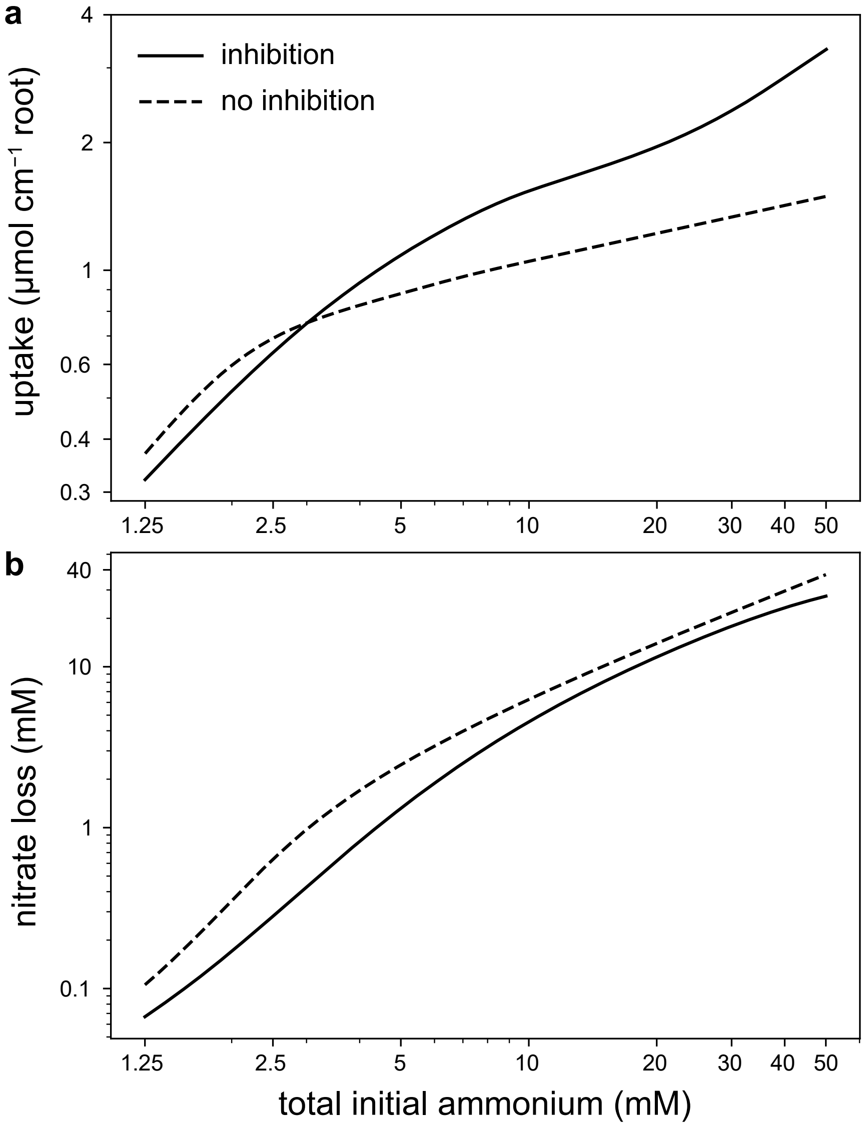

Figure S1: Influence of initial N in soil on (a) total N uptake per root segment of unit length and (b) nitrate concentration loss from the rhizosphere. The ammonium in soil is buffered, i. e., [0.025; 1] mM ammonium in the plant available soil solution. Parameter values are in Table 1. Note, the axes are log-transformed.

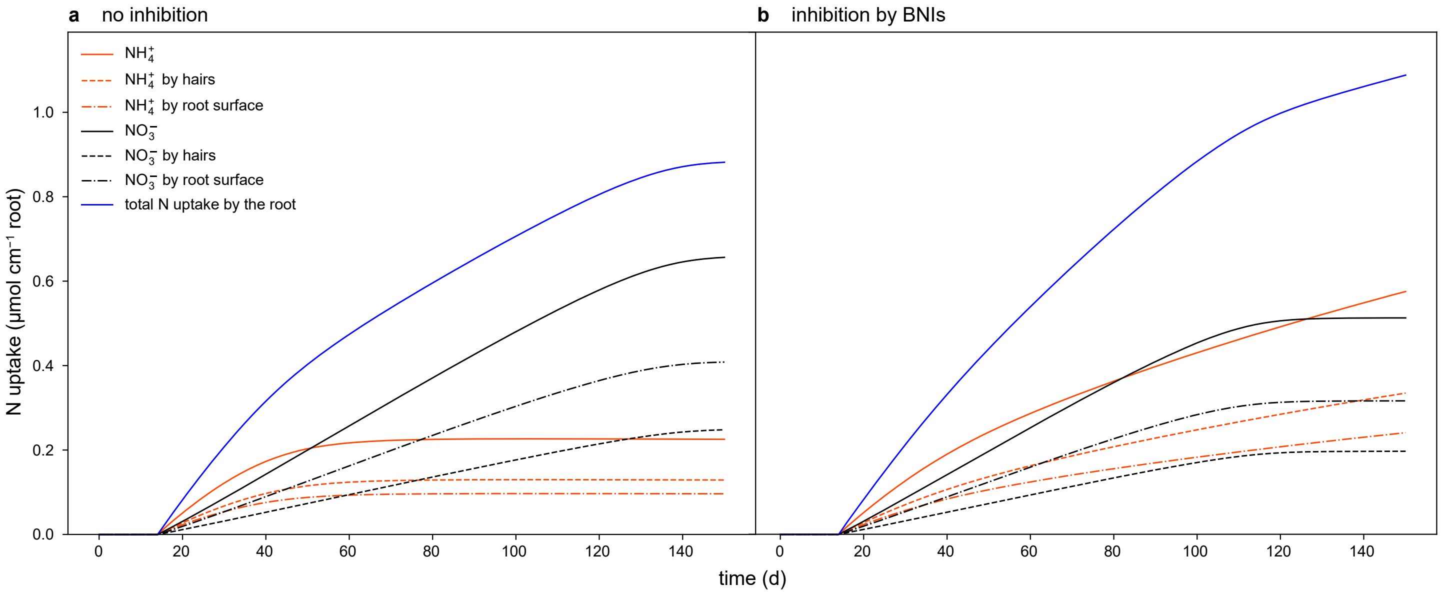

Figure S2: Cumulative uptake of nitrate and ammonium by a root of unit length and its hairs, without (a) and with BNIs (b). Parameter values are in Table 1.
